# Acetazolamide-based fungal chitinase inhibitors

**DOI:** 10.1016/j.bmc.2010.09.062

**Published:** 2010-12-01

**Authors:** Alexander W. Schüttelkopf, Ludovic Gros, David E. Blair, Julie A. Frearson, Daan M.F. van Aalten, Ian H. Gilbert

**Affiliations:** Division of Biological Chemistry and Drug Discovery, College of Life Sciences, University of Dundee, Sir James Black Centre, Dundee DD1 5EH, UK

**Keywords:** Chitinase, *Aspergillus fumigatus*

## Abstract

Chitin is an essential structural component of the fungal cell wall. Chitinases are thought to be important for fungal cell wall remodelling, and inhibition of these enzymes has been proposed as a potential strategy for development of novel anti-fungals. The fungal pathogen *Aspergillus fumigatus* possesses two distinct multi-gene chitinase families. Here we explore acetazolamide as a chemical scaffold for the inhibition of an *A. fumigatus* ‘plant-type’ chitinase. A co-crystal structure of *Af*ChiA1 with acetazolamide was used to guide synthesis and screening of acetazolamide analogues that yielded SAR in agreement with these structural data. Although acetazolamide and its analogues are weak inhibitors of the enzyme, they have a high ligand efficiency and as such are interesting leads for future inhibitor development.

## Graphical abstract

**Figure:**
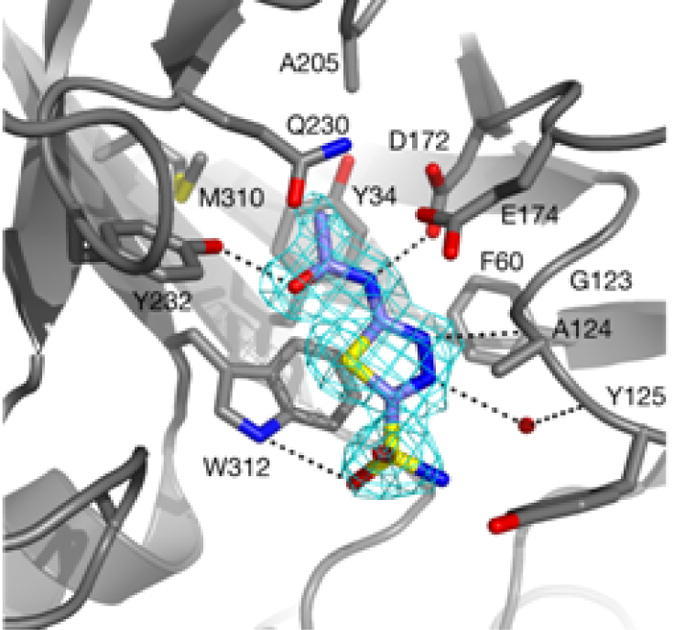

## 1 Introduction

*Aspergillus fumigatus* is the causative agent of aspergillosis, a life-threatening fungal infection that targets a rising population of immunocompromised patients.^1^ Currently available anti-fungal drugs, such as the azoles, amphotericin B and the candins are only partially effective^2,3^ and resistant *Aspergillus* strains have started to appear in hospital settings.^4,5^ Thus there is a need for the identification of novel targets and the development of new anti-fungal agents. Enzymes involved in the biogenesis/turnover of the fungal cell wall are thought to represent possible targets.

Chitin, a polymer of β(1,4)-linked *N*-acetylglucosamine (GlcNAc), is an essential structural component of the fungal cell wall, giving it structural rigidity and chemical/biological stability. Because of the inherent rigidity of chitin, fungi need to partially hydrolyse the chitin layer for cell division and morphogenesis, which is carried out by family 18 chitinases.^6^ Two subclasses of family 18 chitinases exist: the ‘bacterial-type’ chitinases are found in bacteria, fungi and mammals; the ‘plant-type’ chitinases are found exclusively in plants and fungi. Whereas the ‘bacterial-type’ enzymes are invariably secreted and mostly possess exochitinase activity,^7–10^ the ‘plant-type’ chitinases are frequently cell wall associated and possess endochitinase activity. Several studies have shown that these enzymes are involved in yeast mother–daughter cell separation.^11,12^ Because these enzymes are not intracellular, it is possible to explore a wider area of chemical space for inhibitors, as these would not be required to cross membranes. Whilst humans possess two active chitinases,^13,14^ they are of the ‘bacterial-type’, and to date the only inhibitors reported are the large, hydrophilic natural products, allosamidin,^15^ argifin,^16^ argadin^17^ and the rationally designed drug-like inhibitor *C_2_-dicaffeine*.^18^ There are five ‘plant-type’ chitinases genes in the *A. fumigatus* genome (*Af*ChiA1–5), with a currently unknown transcription profile. Sequence alignments show that they have a high degree of structural similarity in the active site, suggesting that it should be possible to design compounds that inhibit all five enzymes.

Recently we have cloned and over-expressed the ‘plant-type’ family 18 chitinase Cts1p from *Saccharomyces cerevisiae* (*Sc*CTS1).^19^ This enzyme was then screened against the Prestwick chemical library of 880 drug-like molecules. From this, three significant hits were identified, 8-chlorotheophylline, acetazolamide and kinetin (Table 1), all of which were competitive inhibitors of the enzyme and were shown to bind in the active site groove, interacting with the catalytic machinery.^19^

Here we describe a study towards the identification of small inhibitor scaffolds (‘fragments’) against the ‘plant-type’ *A. fumigatus* enzyme chitinase A1 (*Af*ChiA1). Two novel *Sc*CTS1 inhibitors, acetazolamide and 8-chlorotheophylline, showed weak inhibition of *Af*ChiA1. We were able to obtain a crystal structure of *Af*ChiA1 in complex with acetazolamide. A number of derivatives of acetazolamide were prepared or purchased and screened against the enzyme *Af*ChiA1; a number were identified with similar activity to acetazolamide.

## 2 Results and discussion

### 2.1 Acetazolamide is an efficient inhibitor of *A. fumigatus* chitinase

Previous work has suggested that the plant-type fungal chitinases may be targets for novel anti-fungal strategies.^11,12,20^ So far the only enzyme from this class characterised in some detail is CST1 from *S. cerevisiae* and the plant enzyme hevamine.^6^ *A. fumigatus* chitinase A1 (*Af*ChiA1) also belongs to the class of plant-type chitinases family, and has been cloned and characterised recently.^21^

**Figure 1:**
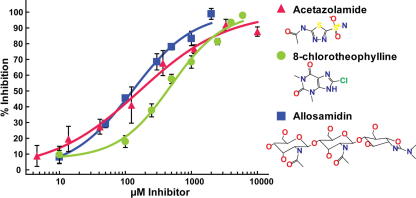
IC_50_ curves determined in triplicate, fitted to a four-parameter logistic dose–response curve (minimum, Hill slope, inflection point and maximum) against *Af*ChiA1 for allosamidin (IC_50_ = 128 μM, Hill slope 0.9), acetazolamide (IC_50_ = 164 μM, Hill slope 1.1) and 8-chlorotheophylline (IC_50_ = 410 μM, Hill slope 1.0) using 4-methylumbelliferyl β-d-*N*,*N′*,*N*″-triacetylchitotrioside (4MU-NAG_3_) as a substrate.

To identify possible inhibitors of *Af*ChiA1, a number of plant-type chitinase inhibitors previously characterised against *Sc*CTS1 were explored as potential scaffolds (Table 1).^19^ Allosamidin is an extensively characterised natural product inhibitor of both plant-type and bacterial-type family 18 chitinases,^15^ and has recently been reported to competitively inhibit *Sc*CTS1 with a *K*_i_ of 0.61 μM^19^ and hevamine with a *K*_i_ of 3.1 μM.^9^ Unfortunately, allosamidin is a substrate analogue with poor drug-like properties (high molecular weight, containing glycosidic bonds and an undesirably low *C* log *P* of −5.2) and the total synthesis is costly and complicated.^23^ Remarkably, allosamidin only weakly inhibits *Af*ChiA1 (IC_50_ = 127 μM, Fig. 1),^21^ which is 30- and 200-fold less potent than values previously reported against hevamine and *Sc*CTS1, respectively. Examination of the purine derivative 8-chlorotheophylline, which had previously been demonstrated to inhibit *Sc*CTS1 with a *K*_i_ of 600 μM,^19^ revealed a similar level of inhibition (IC_50_ = 410 μM, Fig. 1). Two further compounds, kinetin and acetazolamide, have been identified as *Sc*CTS1 inhibitors by screening the Prestwick Chemical Library, with *K*_i_ values of 3.2 μM and 21 μM, respectively.^19^ Kinetin failed to show any discernable effect against *Af*ChiA1 even at concentrations in excess of 1 mM; acetazolamide on the other hand inhibited *Af*ChiA1 with an IC_50_ of 164 μM (Fig. 1), which is an order of magnitude less potent than previously demonstrated against *Sc*CTS1 but not dissimilar to the level of inhibition observed for allosamidin (Fig. 1). It is instructive to compare the ligand efficiencies of the compounds at this stage—that is, the binding energy per non-hydrogen atom.^24^ Due to their small size acetazolamide and 8-chlorotheophylline are the most efficient of these inhibitors (−0.61 and −0.57 kcal mol^−1^ atom^−1^, respectively), compared to allosamidin (−0.19 kcal mol^−1^ atom^−1^). Thus, acetazolamide is a small drug-like molecule that is amenable to preparation of analogues and represents an attractive starting point for further elaboration.

### 2.2 Crystal structure of the *Af*ChiA1–acetazolamide complex suggests possible derivatives

Of the initial leads investigated, acetazolamide was selected as the most promising starting point for the development of *A. fumigatus* plant-type chitinase inhibitors given its high ligand efficiency. *Af*ChiA1 crystals, reported previously^21^, were soaked with acetazolamide, diffraction data were collected to 2.0 Å resolution, and the structure of the *Af*ChiA1–acetazolamide complex was solved by molecular replacement and refined to an *R*_free_ of 0.249 (Table 2) with good stereochemistry. Electron density for the ligand acetazolamide can be seen in both molecules in the asymmetric unit, but it is less clear in chain A, where the active site is partially occluded by a symmetry-related protein molecule. Thus the further discussion of the structure will focus on chain B only, which is less impacted and has a more accessible active site.

**Figure 2:**
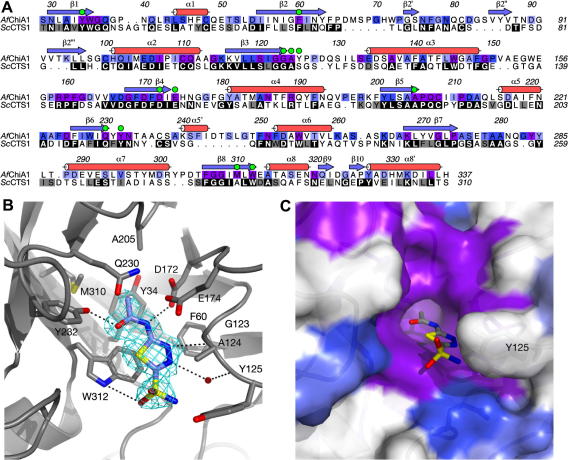
(A) Structure-based sequence alignment of *A. fumigatus* ChiA1 and *S. cerevisiae* CTS1. *Af*ChiA1 secondary structure elements are indicated above the sequence and labelled. Residue numbers are given for *Af*ChiA1. The *Sc*CTS1 sequence is shaded by sequence similarity between the two enzymes shown (black = identical, grey = chemically similar residues), while *Af*ChiA1 is shaded by sequence conservation among *A. fumigatus* ChiA enzymes (purple = 100% identity, then a gradient from blue (mode identical) to white (less identical)). Residues lining the *Af*ChiA1 active site are highlighted by green filled circles. (B) Acetazolamide (slate) binding to the active site of *Af*ChiA1. The protein is shown as a grey cartoon with the side chains of active site residues shown as sticks and labelled. Unbiased (i.e., calculated before the addition of the ligand to the model) *σ*_A_-weighted *F*_o _− *F*_c_ density for acetazolamide contoured at 3.0*σ* is shown in cyan. Possible hydrogen bonds are indicated as black dotted lines, a water participating in indirect hydrogen bonding between ligand and protein is shown as a red sphere. (C) The active site cavity of *Af*ChiA1 (with bound acetazolamide) coloured by similarity among *Af*ChiA proteins as described for panel A. Y125, the only non-conserved residue of the acetazolamide-binding site, is labelled.

The overall binding mode of the ligand to *Af*ChiA1 is essentially identical to that observed for *Sc*CTS1 (Fig. 2B).^19^ The thiadiazole ring stacks with the conserved Trp312, while its ring nitrogens accept hydrogen bonds from the backbone amides of Ala124 and Tyr125, in the latter case indirectly via an active site-bound water molecule. The acetamido group enters, and essentially fills, the small *Af*ChiA1 active site pocket formed by Tyr238, Gln230, Met310, Ala205, Tyr34 and Asp172. It is oriented by two hydrogen bonds, one from its amide to the side chain of Asp172 and one from the Tyr232 side chain hydroxyl to its carbonyl oxygen. The sulfonamide group on the other hand forms few direct interactions with the protein: it accepts a poor hydrogen bond from the Trp312 side chain and otherwise points away from the protein and into the bulk solvent.

The unexpectedly poor inhibition of *Af*ChiA1 by kinetin can be explained by the presence of methionine 310 in the *Af*ChiA1 active site, which replaces an alanine in the corresponding position in *Sc*CTS1 (Fig. 2A), a substitution found in all *A. fumigatus* plant-type chitinases. These residues define the bottom of the active site pocket that accepts the furanyl group of kinetin.^19^ While the pocket is still present in *Af*ChiA1, it is shallower due to the larger Met310 side chain (Fig. 2), rendering it unable to accommodate bulky ligands like kinetin.

*A. fumigatus* is predicted to possess five plant-type GH18 chitinases (*Af*ChiA1–5) that may have overlapping, if not interchangeable, functions. Thus, for an inhibitor to be useful in vivo, it would have to bind effectively to all five *Af*ChiA active sites. The *Af*ChiA1 sequence in Fig. 2A is shaded based on a sequence alignment of *Af*ChiA1–5, indicating residues identical among all five proteins in purple, residues conserved among four or fewer proteins in shades of blue and completely non-conserved residues in white. The same colouring has also been applied to the *Af*ChiA1 active site surface shown in Fig. 2C, demonstrating that, with the exception of the non-conserved but flexible Tyr125,^21^ the part of the active site cleft interacting with acetazolamide is completely conserved among all five *A. fumigatus* plant-type chitinases. This suggests that acetazolamide could bind similarly, both in orientation and in affinity, to these five enzymes. Fig. 2C also highlights additional conserved active site areas that could be used for the further elaboration of the ligand.

To investigate in silico the potential for such elaboration, we used the docking program ligtor^18^ to screen for beneficial substitutions/modifications of either the acetamido or the sulfonamide group, while keeping the rest of the molecule constant. Not surprisingly, the scope for modification at the acetamido group is limited. Docking runs predict that a slight increase in size of this group, for example, by substituting a trifluoroacetamido moiety, could improve overall binding affinity, and even an additional methyl group, yielding a propionamido group, may be tolerated with slight changes to the overall binding mode, but anything larger (including, e.g., isobutyramido groups) cannot be accommodated in the active site pocket and would most likely abolish binding. Modifications/substitutions of the sulphonamide group on the other hand face the opposite problem: as the ligand is essentially pointing away from the active site, most small modifications are tolerated but do not yield additional interactions between ligand and protein. Larger additions to the existing scaffold may be able to interact with additional parts of the *Af*ChiA1 active site, but the required flexibility of such ligands and the corresponding entropy cost associated with orienting the flexible parts on binding to the protein could negate any positive effects on the predicted ligand affinity. To test these computational predictions, a number of acetazolamide derivatives were either synthesised or obtained from commercial suppliers and their binding to *Af*ChiA1 was investigated.

### 2.3 Synthesis and screening of acetazolamide derivatives

**Scheme 1:**
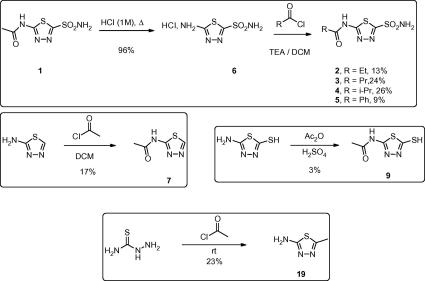
Synthesis of acetazolomide analogues.

As acetozolamide provided an attractive small molecule starting point for a rational focused inhibitor screen, including a structurally defined binding mode, a number of analogues were screened against *Af*ChiA1 (Table 3). The acetazolamide analogues were either synthesised (Scheme 1) or acquired from commercial sources. The synthesis of compounds carried out to is shown in the scheme.

Compounds were screened against *Af*ChiA1 in duplicate. The assay performance statistics generated from screening plates were well within acceptable screening parameters (*Z*′ 0.72 ± 0.04) and the replicate potency determinations correlated well, yielding errors below 45% for all bar one compound (Table 3).

The structures of the compounds allowed determination of the effects of changing the both the sulphonamide (R_1_ in Table 3) and acetamide (R_2_) portions of the molecule. The screen gave a number of compounds with potencies in the 100–500 μM range, that is, similar to the parent compound. A few trends in the SAR can be deduced. Increasing the size of the acetamide moiety by adding an extra methyl (**2**) or a chloro (**20** and **21**) substituent leads to a reduction in activity, while substitution with a trifluoroacetamide group (compare **11** and **15**) is energetically neutral or slightly favourable; this is in accordance with the structural and docking data, as the methyl of the acetamide group essentially fills the active site pocket as described above. At the same time ‘deacetylating’ R_2_ to a free amine also abolishes inhibitory activity (cf. **6**). Replacement of the sulphonamide is generally tolerated: –SH, –Ph, –CF_3_ and –Br substituents as R_1_ (**9**–**12**) produce compounds with similar activity to acetazolamide (**1**). This is perhaps not surprising as the sulphonamide group does not appear to make significant interactions with the protein. Nonetheless the R_1_ substituent does affect affinity as its removal (R_1_ = –H, **7**) or replacement with a methyl (R_1_ = –CH_3_, **8**) again abrogate activity.

## 3 Conclusion

*A. fumigatus* contains five plant-type GH18 chitinases; based on the structural information for *Af*ChiA1, it is predicted that the acetazolamide binding sites of *Af*ChiA1–5 are identical, suggesting it may be possible to develop compounds that inhibit all of these enzymes. We have previously reported various inhibitors of *Sc*CTS1; these showed different inhibition profiles against *Af*ChiA1; in particular the binding pocket which accommodated the acetamide group is much smaller in the case of *Af*ChiA1 compared to *Sc*CTS1. The most promising inhibitor was acetazolamide. Although acetazolamide and various analogues did not show very potent inhibition, they have relatively low molecular weights. Ligand efficiency is a good way to characterise how efficiently these core scaffolds bind and the potential for them to be optimised to low nanomolar compounds.^24^ Some of the compounds (Table 3) have ligand efficiencies of better than −0.3 kcal mol^−1^ atom^−1^. Therefore these possess the potential to be elaborated to compounds with IC_50_ of <10 nM and molecular weight of <500, provided good binding interactions are retained. Most of the interactions with the protein are focused around the amide bond and thiadiazole ring. There is not much scope for further substitution of the acetyl group as the methyl nearly completely occupies a small pocket. However the sulphonamide does not appear to make strong interactions and it is possible to replace this. Therefore optimisation will have to focus on substitution or replacement of this sulphonamide and enhancement of the interactions of the thiadiazole core with the protein.

## 4 Experimental

### 4.1 Expression and purification

*Af*ChiA1 Ser29-Leu335 was expressed and purified as described previously.^21^ Briefly, the enzyme was expressed in *Pichia pastoris* as a secreted protein. The culture supernatant was subjected to dialysis and concentration, then *Af*ChiA1 was purified using anion exchange chromatography followed by gel filtration. The resulting pure *Af*ChiA1 protein was used for both kinetic analysis and crystallization trials.

### 4.2 Crystallisation and structure solution

The protein was concentrated to 36 mg mL^−1^ and crystallized by hanging drop vapour diffusion as described previously.^21^ Acetazolamide was incorporated by adding the solid ligand to a crystal-containing drop and incubating for 30 min at room temperature. After cryoprotection by short immersion in 2.5 M Li_2_SO_4_, data were collected at 100 K on beamline ID14-EH3 at the European Synchrotron Radiation Facility (ESRF, Grenoble, France).

Data were processed and scaled to 2.0 Å using HKL software^26^ and the structure was solved by molecular replacement with AMoRe^27^ using the *Af*ChiA1 apo-structure^21^ as a search model. Refinement of the *Af*ChiA1–acetazolamide complex structure proceeded through rounds of minimisation with REFMAC5^28^ and model building with Coot.^29^ Ligand coordinates and topologies were generated with PRODRG.^30^ PyMol^31^ and ALINE^32^ were used in the preparation of Fig. 2.

### 4.3 *Af*ChiA1 inhibition assays

*Af*ChiA1 activity was assayed in McIlvain’s buffer (pH 5.5).^33^ The final reaction mixture consisted of *Af*ChiA1 (10 nM), 0.05 mg/mL BSA (Thermo) and 4-methylumbelliferyl β-d-*N*,*N*′,*N*″-triacetylchitotrioside (Sigma) (100 μM). Final assay volume was 42 μl in 384-well black polystyrene plates with a final dimethyl sulphoxide (DMSO) concentration of 1% in all samples, including controls. Test and standard compound concentrations ranged from 1000 to 0.15 μM and 10,000 to 1.5 μM, respectively.

Test and standard compounds were placed into columns 1 and 13 of a 384-well polypropylene plate and then serially diluted in 100% DMSO through half log increments using a JANUS 8-channel Varispan automated workstation (PerkinElmer). This produced a compound source plate containing 30 test and 2 standard compounds curves (100 × final assay concentration). From this source plate, 0.42 μl of each compound concentration was then stamped into replicate black 384-well polystyrene assay plates using a Hummingbird (Genomic Solutions).

To the assay plates, 20.8 μl *Af*ChiA1 (20 nM) was added to all wells with the exception the negative controls. The reaction was initiated by the addition of 20.8 μl of (200 μM) 4-methylumbelliferyl β-d-*N*,*N*′,*N*″-triacetylchitotrioside (stock concentration 200 μM), both previous additions were executed using a FlexDrop reagent dispenser (PerkinElmer).

Assay plates were then incubated on a microtitre plate shaker (Heidolph) at room temperature for 70 min. Fluorescence generated from the release of 4-methylumbelliferone was quantified using an Envision 2102 Multilabel Reader (PerkinElmer) equipped with 340 nm excitation (band width 60 nm) and 460 nm emission (band width 25 nm) filters.

ActivtyBase (Abase) version 5.4 from IDBS was used for the data processing and analysis. All curve fitting was undertaken using a 4 Parameter Logistic dose–response curve using XLFit 4.2 Model 205.

### 4.4 Compound Synthesis

#### 4.4.1 Synthesis of 5-amino-2-sulfamoyl-1,3,4-thiadiazole monohydrochloride (**6**)

Hydrochloric acid (70 mL, 70.00 mmol, 5.2 equiv) was added to acetazolamide (2.995 g, 13.34 mmol, 1.0 equiv) and the mixture stirred for 3 h at reflux. The crude material was purified by column chromatography (CHCl_3_/MeOH: 100/0 to 70/30) to yield the product (2.768 g, 96%); mp 179–180 °C; *R*_f_ = 0.26 (CHCl_3_/MeOH: 80/20); *δ*_H_ (500 MHz, DMSO) 7.91 (2H, br s, NH_2_-6), 8.09 (3H, s, NH_3_-7); *δ*_C_ (125 MHz, DMSO) 157.8 (C-2), 171.6 (C-5); *m*/*z* (ES^+^): 181.1 ([M+H−Cl]^+^, 100%); HRMS (ES^+^) 180.9849. ([M+H−Cl]^+^ C_2_H_5_N_4_O_2_S_2_ requires 180.9848).

#### 4.4.2 Synthesis of 5-propylamido-2-sulfamoyl-1,3,4-thiadiazole (**2**)

5-Amino-2-sulfamoyl-1,3,4-thiadiazole monohydrochloride (218 mg, 1.01 mmol, 1.0 equiv) was dissolved in DCM (6 mL). Triethylamine (0.30 mL, 2.16 mmol, 2.1 equiv) was added and the solution stirred for 1.5 h at rt. Then, propionyl chloride (0.20 mL, 2.26 mmol, 2.2 equiv) was slowly added and the mixture left stirring for 1.5 h at rt. Water (1 mL) was added and the precipitate filtered and dried under vacuum. The solid (158 mg) was purified by column chromatography (CHCl_3_/MeOH: 100/0 to 78/22) to yield the product (32 mg, 13%); mp 253–255 °C; *R*_f_ = 0.58 (CHCl_3_/MeOH: 80/20); *δ*_H_ (500 MHz, DMSO) 1.12 (3H, t, H-10, *J* = 7.5), 2.55 (2H, q, H-9, *J* = 7.5), 8.34 (2H, br s, NH_2_-6), 13.00 (1H, br s, NH-7); *δ*_C_ (125 MHz, DMSO) 8.8 (C-10), 28.2 (C-9), 161.2 and 164.1 (C-2 and C-5), 173.0 (C-8); *m*/*z* (ES^+^): 237.0 ([M+H]^+^, 100%), 495.0 ([2M+H]^+^, 71%); HRMS (ES^+^) 237.0101. ([M+H]^+^ C_5_H_9_N_4_O_3_S_2_ requires 237.0111).

#### 4.4.3 Synthesis of 5-butyramido-2-sulfamoyl-1,3,4-thiadiazole (**3**)

5-Amino-2-sulfamoyl-1,3,4-thiadiazole monohydrochloride (286 mg, 1.32 mmol, 1.0 equiv) was dissolved in DCM (7 mL). Triethylamine (0.35 mL, 2.51 mmol, 1.9 equiv) was added and the solution stirred for 1.5 h at rt. Then, butyryl chloride (0.25 mL, 2.36 mmol, 1.8 equiv) was slowly added and the mixture left stirring for 4 h at rt. Water (1 mL) was added and the precipitate filtered and dried under vacuum. The solid (90 mg) was purified by column chromatography (CHCl_3_/MeOH: 100/0 to 78/22) to yield the product (79 mg, 24%); mp 244–246 °C; *R*_f_ = 0.54 (CHCl_3_/MeOH: 80/20); *δ*_H_ (500 MHz, DMSO) 0.91 (3H, t, H-11, *J* = 7.4), 1.65 (2H, sext, H-10, *J* = 7.4), 2.52 (2H, m, H-9), 8.33 (2H, br s, NH_2_-6), 12.99 (1H, br s, NH-7); *δ*_C_ (125 MHz, DMSO) 13.4 (C-11), 17.9 (C-10), 36.7 (C-9), 161.1 and 164.2 (C-2 and C-5), 172.2 (C-8); *m*/*z* (ES^+^): 251.0 ([M+H]^+^, 73%); 523.0 ([2M+H]^+^, 100%); HRMS (ES^+^) 251.0257. ([M+H]^+^ C_6_H_11_N_4_O_3_S_2_ requires 251.0267).

#### 4.4.4 Synthesis of 5-(2-methyl-propylamido)-2-sulfamoyl-1,3,4-thiadiazole (**4**)

5-Amino-2-sulfamoyl-1,3,4-thiadiazole monohydrochloride (274 mg, 1.26 mmol, 1.0 equiv) was dissolved in DCM (7 mL). Triethylamine (0.35 mL, 2.51 mmol, 2.0 equiv) was added and the solution stirred for 1.5 h at rt. Then, isobutyryl chloride (0.25 mL, 2.34 mmol, 1.9 equiv) was slowly added and the mixture left stirring for 2 h at rt. Water (1 mL) was added and the precipitate filtered and dried under vacuum. The solid was purified by column chromatography (CHCl_3_/MeOH: 100/0 to 80/20) to yield the product (90 mg, 26%); mp 254–255 °C; *R*_f_ = 0.65 (CHCl_3_/MeOH: 80/20); *δ*_H_ (500 MHz, DMSO) 1.16 (6H, d, H-10, *J* = 6.9), 2.82 (1H, Sept, H-9, *J* = 6.8), 8.34 (2H, br s, NH_2_-6), 13.01 (2H, br s, NH-7); *δ*_C_ (125 MHz, DMSO) 18.9 (C-10), 33.9 (C-9), 161.3 and 164.3 (C-2 and C-5), 176.1 (C-8); *m*/*z* (ES^+^): 523.0 ([2M+Na]^+^, 100%), 251.0 ([M+H]^+^, 33%); HRMS (ES^+^) 251.0272. ([M+H]^+^ C_6_H_11_N_4_O_3_S_2_ requires 251.0267).

#### 4.4.5 Synthesis of 5-benzylamido-2-sulfamoyl-1,3,4-thiadiazole (**5**)

5-Amino-2-sulfamoyl-1,3,4-thiadiazole monohydrochloride (315 mg, 1.45 mmol, 1.0 equiv) was dissolved in DCM (7 mL). Triethylamine (0.40 mL, 2.87 mmol, 2.0 equiv) was added and the solution stirred for 1.5 h at rt. Then, benzoyl chloride (0.30 mL, 2.56 mmol, 1.8 equiv) was slowly added and the mixture left stirring for 2.5 h at rt. Water (1 mL) was added and the precipitate filtered and dried under vacuum. The solid (317 mg) was purified by column chromatography (CHCl_3_/MeOH: 100/0 to 78/22) to yield the product (36 mg, 09%); mp 260–261 °C; *R*_f_ = 0.66 (CHCl_3_/MeOH: 80/20); (found: C, 38.3; H, 3.2; N, 17.9; S, 21.2. C_9_H_8_N_4_O_3_S_2_·0.7MeOH requires C, 38.0; H, 3.5; N, 18.3; S, 20.9); *δ*_H_ (500 MHz, DMSO) 7.60 (2H, dt, H-11, *J* = 7.8, 1.8), 7.71 (1H, tt, H-12, *J* = 7.4, 1.2), 8.16 (2H, dd, H-10, *J* = 8.3, 1.1), 8.38 (2H, br s, NH_2_-6), 13.55 (1H, br s, NH-7); *δ*_C_ (125 MHz, DMSO) 128.6 and 128 (C-10 and C-11), 130.9 (C-9), 133.4 (C-12), 162.2 and 164.7 (C-2 and C-5), 165.7 (C-8); *m*/*z* (ES^+^): 285.0 ([M+H]^+^, 100%), 569.0 ([2M+H]^+^, 20%); HRMS (ES^+^) 285.0102. ([M+H]^+^ C_9_H_9_N_4_O_3_S requires 285.0111).

#### 4.4.6 Synthesis of 5-acetamido-1,3,4-thiadiazole (**7**)

2-Amino-1,3,4-thiadiazole (161 mg, 1.54 mmol, 1.0 equiv) was dissolved in DCM (2 mL). Acid chloride (0.15 mL, 2.11 mmol, 1.4 equiv) was slowly added (exothermic), and the mixture stirred for 5 h at rt. The solution was concentrated after addition of H_2_O (1 mL). The crude product was purified by column chromatography (CHCl_3_/MeOH: 100/0 to 90/10) to yield the product (38 mg, 17%); mp 276–277 °C; *R*_f_ = 0.83 (CHCl_3_/MeOH: 80/20); (found: C, 33.9; H, 3.4; N, 27.6; Cl, 21.8. C_4_H_5_N_3_OS·0.03HCl·0.18MeOH requires C, 33.5; H, 3.8; N, 28.0; S, 21.4); *δ*_H_ (500 MHz, DMSO) 2.20 (3H, s, H-8), 9.15 (1H, s, H-2), 12.55 (1H, br s, NH-6); *δ*_C_ (125 MHz, DMSO) 22.4 (C-8), 148.5 (C-5), 158.5 (C-2), 168.6 (C-7); *m*/*z* (ES^+^): 144.0 ([M+H]^+^, 60%), 166.0 ([M+Na]^+^, 100%); HRMS (ES^+^) 144.0226. ([M+H]^+^ C_4_H_6_N_3_OS requires 144.0226).

#### 4.4.7 5-Acetamido-2-thiol-1,3,4-thiadiazole (**9**)

5-Amino-1,3,4-thiadiazole-2-thiol (554 mg, 4.08 mmol, 1.0 equiv), acetic anhydride (1.8 mL, 19.08 mmol, 4.7 equiv) and concd sulphuric acid (20 mL, 0.37 mmol, 0.09 equiv) were stirred for 30 min on a steam bath. After cooling, the mixture was concentrated under vacuum, and then purified by column chromatography (CHCl_3_/MeOH: 100/0 to 70/30) to yield the product (22 mg, 03%); mp 293–295 °C; *R*_f_ 0.24 (CHCl_3_/MeOH: 90/10); (found: C, 28.1; H, 3.0; N, 22.5; S, 35.1. C_4_H_5_N_3_OS_2_·0.3MeOH requires C, 27.9; H, 3.4; N, 22.7; S, 34.7); *δ*_Η_ (500 MHz, DMSO) 2.14 (3H, s, H-9), 12.45 (1H, br s, NH-7), 14.06 (1H, br s, SH-6); *δ*_C_ (125 MHz, DMSO) 22.3 (C-9), 152.2 (C-5), 169;4 (C-8), 183.5 (C-2); *m*/*z* (ES^+^): 176.0 ([M+H]^+^, 100%); HRMS 175.9946. ([M+H]^+^ C_4_H_6_N_3_OS_2_ requires 175.9947).

#### 4.4.8 5-Amino-2-methyl-1,3,4-thiadiazole (**19**)

Acetyl chloride (0.50 mL, 7.04 mmol, 2.2 equiv) was slowly added to thiosemicarbazide (291 mg, 3.16 mmol, 1.0 equiv) and the mixture stirred for 4 h at rt. A solution of NaOH 50% was added till pH 12–14, and the mixture concentrated under vacuum. The crude material was purified by column chromatography (CHCl_3_/MeOH: 100/0 to 91/09) to yield the product (85 mg, 23%); mp 273–274 °C; *R*_f_ = 0.79 (CHCl_3_/MeOH: 80/20); (found: C, 31.9; H, 4.4; N, 35.4; S, 27.4. C_3_H_5_N_3_S·0.05AcOH requires C, 31.5; H, 4.4; N, 35.6; S, 27.1); *δ*_Η_ 500 MHz, DMSO 2.17 (3H, s, H-7), 13.15 (2H, br s, NH_2_-6); *δ*_C_ (125 MHz, DMSO) 10.8 (C-7), 148.8 (C-5), 165.8 (C-2); *m*/*z* (ES^+^): 115.8 ([M+H]^+^, 100%); 253.2 ([2M+Na]^+^, 100%); HRMS (ES^+^) 116.0275. ([M+H]^+^ C_3_H_6_N_3_S requires 116.0277).

## Figures and Tables

**Table 1:** Inhibitors of *Sc*CTS1 and their activity against *Af*ChiA1

Name	Allosamidin	8-Chloro-theophylline	Acetazolamide	Kinetin
Structure				
*Sc*CST1 *K*_i_	0.61	340	21	3.2
*Af*ChiA1 IC_50_	127	410	164	>1000
hCHT IC_50_	0.04	>2500	>1000	>1000

All data given in μM. hCHT is human chitinase. Data for *Sc*CST1 have been reported previously.^19^ The IC_50_ of allosamidin against hCHT has been reported previously.^22^

**Table 2:** X-ray diffraction/refinement statistics for the *Af*ChiA1–acetazolamide complex

Resolution range (Å)	20.00–2.00 (2.05–2.00)
Number of observed reflections	27,0663
Number of unique reflections	67,879 (4270)
Completeness (%)	98.1 (92.9)
Redundancy	4.0 (3.5)
*I*/*σ*(*I*)	13.5 (2.5)
*R*_merge_	0.085 (0.522)
Wilson *B* (Å^2^)	22.5
*R*_work_, *R*_free_	0.216, 0.249
Bond length rmsd from ideality (Å)	0.017
Bond angle rmsd from ideality (°)	1.5
<*B*>, overall (Å^2^)	27.2
<*B*>, protein (Å^2^)	26.5
<*B*>, solvent (Å^2^)	34.1
<*B*>, ligand (Å^2^)	32.1
Ramachandran plot	
Most favoured (%)	88.6
Additionally allowed (%)	10.8
Generously allowed (%)	00.2

Values in parentheses pertain to the highest resolution shell. Ramachandran plot statistics were calculated with PROCHECK.^25^

**Table 3:** Activity of compounds investigated

Compd			IC_50_ (μM)	Hill slope	L.E.	% inhibition at 1 mM
	R^1^	R^2^				
1	–SO_2_NH_2_	–NHCOCH_3_	164 ± 75	1.1	−0.40	88
2	–SO_2_NH_2_	–NHCOCH_2_CH_3_	315 ± 65	0.71	−0.34	76
3	–SO_2_NH_2_	–NHCO(CH_2_)_2_CH_3_	>1000			33
4	–SO_2_NH_2_	–NHCOCH(CH_3_)_2_	>1000	N/A		N/A
5	–SO_2_NH_2_	–NHCOPh	850 ± 74	0.5	−0.23	51
6	–SO_2_NH_2_	–NH_2_	>1000	N/A		N/A
7	–H	–NHCOCH_3_	>1000	N/A		25
8	–Me	–NHCOCH_3_	>1000	N/A		N/A
9	–SH	–NHCOCH_3_	730 ± 120	1.1	−0.43	64
10	–Ph	–NHCOCH_3_	479 ± 210	0.5	−0.30	60
11	–CF_3_	–NHCOCH_3_	141 ± 210	1.1	−0.44	91
12	–Br	–NHCOCH_3_	243 ± 98	0.7	−0.65	76
13		–NHCOCH_3_	>1000	N/A		22
14		–NHCOCH_3_	>1000			38
15	–Ph	–NHCOCF_3_	320 ± 60	1.2	−0.26	85
16	Morpholino	–NHCOCF_3_	>1000	N/A		22
17		–NHCOCF_3_	>1000	N/A		30
18	–Et	–NHCOCH_2_CH_3_	>1000	N/A		18
19	–Me	–NH_2_	>1000	N/A		21
20	–CH_2_CH(CH_3_)_2_	–NHCOCH_2_Cl	>1000	N/A		N/A
21	–CF_3_	–NHCOCH_2_Cl	>1000	N/A		N/A
22	–CF_3_	–CO(CH_2_)_2_CO_2_H	>1000	N/A		N/A
Allosamidin			127		−0.12	
8-Chloro-theophylline			410		−0.33	
Kinetin			>1000			

L.E. = ligand efficiency in kcal mol^−1^ atom^−1^.^24^ This was calculated from the equation: Δ*G* = −RT ln(1/IC_50_). IC_50_ standard deviations calculated with 95% confidence limit.
